# The Rarity of Survival to Old Age Does Not Drive the Evolution of Senescence

**DOI:** 10.1007/s11692-016-9385-4

**Published:** 2016-05-04

**Authors:** Maarten J. Wensink, Hal Caswell, Annette Baudisch

**Affiliations:** 1 Max Planck Odense Center on the Biodemography of Aging, Odense, Denmark; 2 Institute of Public Health, University of Southern Denmark, Winsløws Vej 9B, 5000 Odense C, Denmark; 3 Max Planck Research Group on Modeling the Evolution of Aging, Max Planck Institute for Demographic Research, Konrad Zuse Strasse 1, 18055 Rostock, Germany; 4 Leyden Academy of Vitality and Ageing, Poortgebouw LUMC, Rijnsburgerweg 10, 2333 AA Leiden, The Netherlands; 5 Institute for Biodiversity and Ecosystem Dynamics, University of Amsterdam, 1098 XH Amsterdam, The Netherlands; 6 Biology Department MS-34, Woods Hole Oceanographic Institution, Woods Hole, MA 02543 USA; 7 Biology Department, University of Southern Denmark, Campusvej 55, 5230 Odense M, Denmark

**Keywords:** Extrinsic mortality, Survivorship, Age distribution, Selection gradient, Senescence

## Abstract

The evolution of senescence is often explained by arguing that, in nature, few individuals survive to be old and hence it is evolutionarily unimportant what happens to organisms when they are old. A corollary to this idea is that extrinsically imposed mortality, because it reduces the chance of surviving to be old, favors the evolution of senescence. We show that these ideas, although widespread, are incorrect. Selection leading to senescence does not depend directly on survival to old age, but on the shape of the stable age distribution, and we discuss the implications of this important distinction. We show that the selection gradient on mortality declines with age even in the hypothetical case of zero mortality, when survivorship does not decline. Changing the survivorship function by imposing age independent mortality has no affect on the selection gradients. A similar result exists for optimization models: age independent mortality does not change the optimal result. We propose an alternative, brief explanation for the decline of selection gradients, and hence the evolution of senescence.

## Introduction

As old as the evolutionary theory of senescence is its underlying and widespread tenet that senescence evolves because survivorship dwindles with age. Consequently, higher mortality should lead to more senescence. In contrast, several authors have indisputably shown over the last decades that this logic is incorrect (Abrams 1993; Caswell 2007; Moorad and Promislow 2010; Caswell and Shyu 2016). Yet, these results did not suffice to erase the prevailing misconception (recent examples include Nussey et al. 2008; Vijg and Campisi 2008; Monaghan et al. 2008; Fabian and Flatt 2011, 2012; Regan and Partridge 2013; Gems and Partridge 2013), which is problematic, because empirical studies keep on testing a theoretical prediction (e.g. Chen and Maklakov 2012; Reznick et al. 2004; Stearns et al. 2000; see review in Williams et al. 2006) that is, as such, not predicted. What to do, when something repeatedly proven to be wrong is still taken to be right? Challenging the insight of Max Planck who said that “Science advances one funeral at a time”, here we attempt to advance understanding of the evolutionary theories of aging conditional on survivorship.

The issue is obvious and at the same time tricky. That is why confusion may persist so successfully. Clearly, survivorship falls over age, and clearly adding some fixed amount of mortality at every age makes survivorship fall off more steeply. But such a shift in mortality merely reduces fitness; it does not change the selection gradients over the fitness landscape (Caswell 2007; Caswell and Shyu 2016). The selection gradients still decline following the same pattern as in the absence of such mortality; selection does not favor young over old ages more or less strongly than before.

In this paper we aim to go through the issue methodically. We show how selection gradients depend on survivorship only indirectly. We explore various scenarios that we think clarify the topic: a scenario with zero mortality, such that survivorship does not decline (but selection gradients do), a scenario in which survivorship is changed by adding an age independent term to mortality (leaving selection gradients unchanged), and a scenario in which we show that the results of optimization models are independent of such a fixed, age independent mortality component. We propose a brief, more correct basic explanation of why selection gradients decline.

## Evolution of Senescence: Selection Gradients

### Why Senescence Can Evolve: Selection Gradients Decline with Age

Selection on a trait depends on the sensitivity of fitness to a change in that trait, the so called selection gradient.^1^ Senescence can evolve because selection gradients on mortality and fertility decline with age (Hamilton 1966). Darwinian fitness is given by *r* (Fisher 1930; Lande 1982; Metz et al. 1992; Charlesworth 1994), the unique real root of the Euler-Lotka equation (Lotka 1924):

1 $$\begin{aligned} \int _0^{\infty } e^{-rx}\ell (x)m(x)dx=1. \end{aligned}$$

Here, *m*(*x*) is the reproductive rate at age *x*, while $\ell (x)$ denotes survivorship up to age *x*, which is a function of the mortality rate $\mu (x)$:

2 $$\begin{aligned} \ell (x)=e^{-\int _0^x \mu (t)dt}. \end{aligned}$$

The response of fitness *r* to changes in mortality and fertility across ages is given by the differential

3 $$\begin{aligned} dr=\int _0^{\infty } \left[ H^*(a) dm(a) + H^{\dagger }(a) d\mu (a) \right] da, \end{aligned}$$

where

4 $$\begin{aligned} H^*(a)= & {} \frac{1}{T} e^{-ra}\ell (a) \end{aligned}$$

5 $$\begin{aligned} H^{\dagger }(a)= & {} -\frac{1}{T} \int _{a}^{\infty } e^{-rx}\ell (x)m(x)dx, \end{aligned}$$

with

6 $$\begin{aligned} T=\int _0^{\infty } xe^{-rx}\ell (x)m(x)dx \end{aligned}$$

being generation time (Wensink et al. 2014a). $H^*(a)$ and $H^{\dagger }(a)$ are the selection gradients on age-specific fecundity and mortality respectively, originally derived by Hamilton (1966). Observing that the absolute values of (4) and (5) decline with age for all life histories^2^, any evolutionary (dis)advantage later in life is correspondingly devaluated. Hence, the cost of any age related deterioration is also limited.

### Why Selection Gradients Decline with Age

Hamilton’s expressions in (4) and (5) can be reformulated (Caswell 1978, 2010) to reveal what drives the decline in selection gradients with age. Let *v*(*a*) be the reproductive value, which quantifies the value of the expected reproductive contribution of an organism that is alive and of age *a*:

7 $$\begin{aligned} v(a)=\frac{e^{ra}}{\ell (a)}\int _a^{\infty } e^{-rx}\ell (x)m(x)dx. \end{aligned}$$

Let *c*(*a*) be the stable age distribution, which gives the proportional composition of the population by age:

8 $$\begin{aligned} c(a)=\frac{e^{-ra}\ell (a)}{\int _0^{\infty } e^{-rx}\ell (x)dx}. \end{aligned}$$

Finally, let *b* be the birth rate:

9 $$\begin{aligned} b=\left[ \int _0^{\infty } e^{-rx}\ell (x)dx \right] ^{-1}. \end{aligned}$$

With *T* as in Eq. (6), Hamilton’s indicators of selection pressure can be written as:

10 $$\begin{aligned} H^*(a)= & {} \frac{c(a)}{bT} \end{aligned}$$

11 $$\begin{aligned} H^{\dagger }(a)= & {} \frac{- c(a)v(a)}{bT}. \end{aligned}$$

The denominator *bT* is independent of age and simply scales the value of selection gradients.

The numerator of each indicator contains the proportional abundance of organisms (Eq. 8) weighted by the reproductive value of the next age class (Eq. 7). Since the reproductive value of the initial age class equals $v(0)=1$ (Eq. 1), the abundance of mothers at age *a* in Eq. (10) is weighted by one, i.e. the reproductive value of newborns. The negative sign in $H^{\dagger }$ reflects that increasing mortality affects fitness negatively.

The decomposition of selection gradients in Eqs. (10) and (11) into proportional abundance of individuals and their reproductive value clarifies why the rarity of survival to old age does not suffice to explain the decline in selection gradients and, hence, the evolution of senescence. Reproductive value (Eq. 7) is weighted not by the probability of surviving to age *a*, but by the proportional abundance of organisms of age *a* in the stable age distribution, *c*(*a*). This abundance depends not only on survivorship $\ell (a)$, but also on the inflow of newborns into the population. If many young organisms are added to a population, the proportion of older organisms is correspondingly reduced, irrespective of their mortality. The term $e^{-ra}$ in Eq. (8) models this effect on the age composition in a stable population.

The difference between survivorship and the stable age distribution is vital, as we illustrate in a hypothetical counter-example. Suppose that mortality was completely eliminated, so that survivorship did not decline with age. What would happen to the selection gradients?

If mortality were zero at all ages, survivorship would remain constant at one:

12 $$\begin{aligned} \left. \;\ell (a)\, \right| _{\mu =0}\; \equiv \;1. \end{aligned}$$

The stable age distribution, in contrast, would still change with age and becomes

13 $$\begin{aligned} \left. \;c(a)\,\right| _{\mu =0}\;=\;\frac{e^{-ra}}{\int _0^{\infty } e^{-rx}dx}. \end{aligned}$$

Any reproduction, i.e. $m>0$, implies *r* greater than zero. Hence *c*(*a*) falls with age. Since births add zero-year-olds to the population, this age-class will always be the largest compared to progressively older ages. The stable age distribution declines with age as a result of reproduction, while survivorship remains unchanged.

Hamilton’s selection gradients for the case of zero mortality are

14 $$\begin{aligned} \left. \;H^*(a)\,\right| _{\mu =0}= & {} \frac{e^{-ra}}{T}, \end{aligned}$$

15 $$\begin{aligned} \left. \;H^{\dagger }(a)\,\right| _{\mu =0}= & {} -\frac{1}{T} \int _{a}^{\infty } e^{-rx}m(x)dx. \end{aligned}$$

Provided that *r* is positive, which is the case if there is any reproduction, $H^*(a)$ and $H^{\dagger }(a)$ still decline with age, even though survivorship does not.

In this hypothetical, mortality-free situation, declining survivorship is absent. And yet we find that selection gradients decline with age, because young organisms abound, which is entirely due to reproduction that fuels population growth as modeled in *c*(*a*). Hence declining survivorship is not a prerequisite for declining gradients.

In a (more realistic) scenario with non-zero mortality, selection gradients remain unaffected by imposing age-independent mortality (Abrams 1993; Caswell 2007; Monaghan et al. 2008; Caswell and Shyu 2016). To see this, note that survivorship becomes the product of two exponential functions, one containing a constant that represents age independent (extrinsic) mortality, say $\gamma$, and the other containing all age dependent mortality terms, $\mu _{0}(x)$:

16 $$\begin{aligned} \ell (x)=e^{-\gamma x} e^{-\int _0^x \mu _{0}(t)dt}. \end{aligned}$$

Inserting this description of $\ell (x)$ into the Euler-Lotka Eq. (1) and merging $e^{-rx}$ with $e^{-\gamma x}$ yields

17 $$\begin{aligned} \int _0^{\infty } e^{-(r+\gamma )x}e^{-\int _0^x \mu _{0}(t)dt}m(x)dx=1. \end{aligned}$$

For any specified pattern of reproduction *m*(*x*) and age dependent mortality $\mu _{0}(x)$ there exists one and only one real $r+\gamma$ that satisfies (Eq. 17). If now $\gamma$ is increased, *r* is decreased by exactly the same amount:

18 $$\begin{aligned} \frac{\partial r}{\partial \gamma } = -1. \end{aligned}$$

This effect is observed wherever survivorship and $e^{-rx}$ are multiplied together, as is the case for the stable age distribution *c*(*a*), reproductive value *v*(*a*), generation time *T* and the birth rate *b*, i.e. for all components of the selection gradients [see decompositions (10) and (11)] and therefore for the selection gradients themselves.

In sum, we highlight that, first, selection gradients depend on survivorship only indirectly via the stable age distribution (Eqs. 10 and 11). Second, even if survivorship did not decline with age, the stable age distribution and selection gradients would still decline with age due to reproduction that fuels population growth. And third, that selection gradients do not change if survivorship is changed by the addition of age independent mortality.

## Evolution of Senescence: Optimization

Our considerations so far have focused on selection gradients, which describe how fitness would respond to a change in a trait, and thus predict the direction of evolutionary change in the trait. But they do not specify evolutionary endpoints. An alternative approach that does so is optimization: given mechanistic considerations, what strategy maximizes fitness, and would this strategy favor senescence or not? For example, in the disposable soma theory organisms allocate resources between the competing demands of somatic maintenance (which slows senescence) and reproduction (Kirkwood 1977; Kirkwood and Holliday 1979; Kirkwood and Rose 1991). There are two places to invest: to keep yourself going, or to create more copies of yourself. Resources invested in one function cannot be invested in the other. Depending on the return on each investment, some allocation strategy will maximize fitness, i.e. be optimal.

It has been claimed that the optimal allocation strategy is always one where some amount of resources is invested in reproduction at the cost of some degree of senescence, i.e., the claim that senescence is always evolutionarily optimal (Kirkwood 1977; Kirkwood and Rose 1991; Kirkwood and Austad 2000). The argument is that survivorship is a necessarily decreasing function of age because of extrinsic mortality. Hence, it is argued, investment in somatic maintenance to an extent that it would bring senescence to a halt would merely be wasteful, and resources are better invested in offspring (Kirkwood 1977; Kirkwood and Rose 1991; Kirkwood and Austad 2000). In the same vein, higher levels of extrinsic mortality would correlate with higher rates of senescence. However, if mortality is really extrinsic, would it not affect the offspring as well as the focal organism, and could the argument not be reversed: extrinsic mortality kills offspring, so it would be better to invest in somatic maintenance at a cost to reproduction? This issue clearly needs formalization. Indeed, below we show that the optimum of trade-off models is not affected by an age-invariant mortality term, in line with earlier results (Gadgil and Bossert 1970; Taylor et al. 1974; Law 1979).

Let mortality and fertility be functions of a lower level parameter $\theta$ that is optimized so as to maximize *r*. In addition to $\theta$, *r* depends on age-independent mortality $\gamma$. By definition, this mortality cannot be influenced or avoided by any strategy $\theta$. Given relationship (18), which is a general result of the Euler-Lotka equation (Eq. 1), it follows that

19 $$\begin{aligned} r(\theta ,\gamma )=r(\theta ,0)-\gamma \end{aligned}$$

As $\gamma$ does not depend on $\theta$ it follows that

20 $$\begin{aligned} \frac{dr(\theta ,\gamma )}{d \theta }=\frac{dr(\theta ,0)}{d \theta } \end{aligned}$$

Thus, if $\hat{\theta }$ satisfies the optimality condition

21 $$\begin{aligned} dr= \left. \frac{\partial r(\theta ,0)}{d \theta } \right| _{\theta =\hat{\theta }}=0 \end{aligned}$$

it also satisfies the optimality condition

22 $$\begin{aligned} dr= \left. \frac{\partial r(\theta ,\gamma )}{d \theta } \right| _{\theta =\hat{\theta } }=0 \end{aligned}$$

In words, the optimal value of $\theta$ is independent of (a change in) age independent mortality $\gamma$. Just like survivorship can be changed without affecting the selection gradients, it can also be changed without affecting the optimal solution to a trade-off model. Naturally, mortality may not be age independent, or density dependence may give age dependent effects of age independent mortality. If, however, such is assumed to be the case, this needs to be made explicit.

## Discussion

When it comes to understanding why we age, the rarity of survival to old age alone has long served as *the* explanation for declining selection gradients. This seems curious, because life is driven by birth and death together. Why should one side—survivorship—suffice to explain fundamental patterns of life, such as aging? With the derivations above we have demonstrated that reproduction plays an important role. Births keep on adding new individuals to the population, fueling a population growth factor that reduces the share of old organisms in the population. Even in the absence of death, as we demonstrate, births are enough to achieve declining selection gradients. Mortality is not the all-important driver of selection gradients.

We need a rigorous and lasting shift in understanding what role survivorship plays in affecting optimal life history strategies and selection gradients. The reasoning that selection gradients decline, and senescence evolves, because of declining survivorship is incorrect. Whether a change at some age affects evolution to a smaller or larger degree hinges not on survivorship per se, but on the relative abundance of individuals and their reproductive values. Provided the population is non-decreasing, the stable age distribution is always dominated by younger individuals over older individuals as a result of reproduction. This is true even in the hypothetical case of zero mortality. Survivorship can be changed by an age independent mortality term without affecting the selection gradients. Similarly, changes in age independent mortality leave optimal strategies unaffected. Age *dependent* changes in mortality, on the other hand, may either increase or reduce the tendency to senescence (Caswell and Shyu 2016).

Thinking about Hamilton’s original formulation of selection gradients (Hamilton 1966) in terms of survivorship alone is misleading. An accurate intuition would argue that older organisms have already produced a larger share of their total lifetime reproduction. Therefore a progressively smaller proportion of total production is affected by anything that happens to the focal organism at higher ages, and the focal organism will already have passed on its genes (Flatt and Promislow 2007).

The arguments laid out in this paper have theoretical and practical consequences. Empirical research has shown little support for the “central prediction” of the evolutionary theory of senescence (Williams et al. 2006; Chen and Maklakov 2012), that a higher level of extrinsic mortality (predators, harsh environments, laboratory manipulations) should lead to a higher rate of senescence (Williams et al. 2006; Reznick et al. 2004). A number of authors have called for a more involved theory of senescence, in which mortality is state dependent, and/or in which density effects play a prominent role (e.g. Chen and Maklakov 2012; Williams et al. 2006; Reznick et al. 2004). The results derived here and elsewhere (Abrams 1993; Caswell 2007; Caswell and Shyu 2016) make clear why there is little support for the central prediction. It is not just that this prediction is not born out in biological reality; life history theory simply makes no such prediction. After decades of theoretical work, we are still challenged to develop theory that provides more than an incidental match with the data. Our results corroborate the need for theory that is more involved; it may include combinations of age- and stage-specific mortality (Caswell and Salguero-Gómez 2013), density effects (Abrams 1993), and/or interaction mortality (Caswell 2007). Such a theory should involve mechanisms of senescence, as evolutionary pressures alone are only half the story (Wensink et al. 2014a, b; Baudisch and Vaupel 2012).

## Footnotes

1 In earlier literature, terms like ‘force of selection’ (e.g., Medawar 1952; Hamilton 1966) or ’selection pressure’ (e.g., Emlen 1970) were used for this quantity. Analogies to forces, or pressures, however, obscure the nature of the term as the slope, or gradient, of fitness as a function of the trait. The term was carefully defined by Lande (1982) and Arnold and Wade (1984), and is fundamental to quantitative genetics. It also appears in the formalism of the canonical equation of Adaptive Dynamics (e.g., Doebeli 2011).

2 One special exception must be mentioned. Unlike the gradient on mortality, the selection gradient on fertility can *increase* with age in a declining population. If *r* is sufficiently negative relative to survival probability (Mertz 1971; Caswell and Hastings 1980; Caswell 1982), the stable age distribution, and thus the selection gradient on fertility, will increase with age. It is unlikely that a population would persist in such a negative growth phase for long enough for evolution to act. However, Mertz (1971) suggested that the delayed onset of reproduction in the California condor (*Gymnogyps californianus*) might reflect millenia of population decline from a distribution over all of North America to the species current restricted range in central California. Caswell (1982) proposed that selection gradients while declining could be important for nonequilibrium populations.
